# Salivary cytokines as biomarkers of oral cancer: a systematic review and meta-analysis

**DOI:** 10.1186/s12885-021-07932-3

**Published:** 2021-02-27

**Authors:** Mayara Martina Abatti Chiamulera, Caroline Biazzolo Zancan, Aline Pertile Remor, Marcos Freitas Cordeiro, Frederico Omar Gleber-Netto, Antuani Rafael Baptistella

**Affiliations:** 1 Universidade do Oeste de Santa Catarina (UNOESC), Joacaba, SC Brazil; 2 Programa de Pos-Graduacao em Biociencias e Saude/Universidade do Oeste de Santa Catarina, Joacaba, SC Brazil; 3 Hospital Universitario Santa Terezinha, Joacaba, SC Brazil; 4 Department of Head and Neck Surgery, The University of Texas MD Anderson Cancer Center, Houston, TX USA

**Keywords:** Oral cancer, Head and neck cancer, Saliva, Cytokines, Biomarkers

## Abstract

**Background:**

Oral cancer (OC) is usually diagnosed at advanced clinical stages due to its asymptomatic nature and absence of pathognomonic signs in its early development phase. Delayed diagnosis is one of the major causes of OC treatment failure and poor prognosis. Development of alternative diagnostic approaches are imperative for improving early detection and therapeutic success rates. Salivary cytokines (SC) have been studied as potential diagnostic biomarkers for OC and may represent a potential tool for improvement of its early detection.

**Methods:**

In this systematic review and meta-analysis we identified SC studied as OC biomarkers by systematically reviewing the PubMed and Cochrane Library databases using the terms: “*oral cancer”*, “*cytokine”*, and *“saliva”*, and also combined with “*interleukin”* or “*interferon”*. Only case-control studies that measured SC by ELISA from treatment naïve patients were included in the qualitative review. For the meta-analysis were included all comparable studies that provided enough data (sample size, mean and standard deviation or standard error of the mean) for SC levels in OC patients, non-cancer controls and patients with oral potentially malignant disorders (OPMD), including leukoplakia. Comparisons with patients with oral lichen planus (OLP) and gingivitis were included in the qualitative analysis.

**Results:**

A total of 28 articles (from 2004 to 2018) were included in the systematic review, describing 10 different SC, being IL-8 and IL-6 the most studied ones. SC levels were consistently higher among OC patients when compared to healthy controls and to patients with OPMD, OLP and gingivitis. Meta-analysis including 23 eligible studies showed that IL-8, IL-6, TNF-α, IL-1β and IL-10 salivary levels were significantly higher in OC patients compared to controls; and that IL-8, IL-6, TNF-α and IL-1β salivary levels were also higher in OC patients compared to individuals with OPMD. When compared to healthy controls, OPMD patients showed significantly higher IL-6 and TNF-α salivary levels.

**Conclusions:**

Our analyses showed that the salivary levels of some cytokines are consistently different among OC, OPMD and healthy patients, indicating that these SC may represent potential diagnostic biomarkers for OC and OPMD. Despite of that, SC levels were highly variable among studies, suggesting that further technical improvement and standardization for SC measurement by ELISA is needed in order to successfully translate these biomarkers to the clinical practice.

## Background

Oral squamous cell carcinoma (OC) is the most common type of oral cancer, and represents ~ 2% of all cancer cases in the USA. More than 50% of OC patients are diagnosed at advanced clinical stage, with large primary tumors and nodal or distant dissemination. Patients with advanced OC are usually treated with multimodal therapeutic approaches, which are poorly effective and linked to high morbidity [1, 2]. The 5-year survival rate for advanced OC patients is around 50%, while 85% survival rate is observed for early-stage patients [3, 4]. Thus, early diagnosis is a major prognostic factor for OC patients.

Unfortunately, diagnosis of OC at early stages is challenging. Detection of early lesions is usually incidental, since they are usually asymptomatic and rarely perceived by patients. Clinically, early OC lesions may resemble other benign oral mucosal conditions, which can lead to delayed diagnosis. Also, many of the OC cases are preceded by lesions with considerable potential for malignant transformation (mostly oral leukoplakia), which are collectively named as oral potentially malignant disorders (OPMD). Although only 2% of the OPMD will eventually turn into cancer, determination of its malignant transformation risk and distinction from early stage OC are quite challenging [4–7]. Thus, histopathological assessment is imperative for definitive diagnosis which can contribute to further delay in OC detection [8, 9].

Considering that, many research groups have been seeking for early OC detection biomarkers, and differentiate it from benign lesions with similar clinical features. Because saliva is in intimate contact with the oral mucosa, it has been widely studied as a source of OC biomarkers [10]. Saliva carries molecules and cells originated in the aero-digestive tract, as well as nucleic acids and proteins that are passively and actively transported from the circulatory system into the salivary glands [11]. In this way, it has been suggested that saliva has a similar potential as a source of biomarkers as the blood, but with the advantage of being obtained by non-invasive, inexpensive, and safer techniques [12].

Several exploratory case-control studies found that salivary cytokines (SC) were highly deregulated in OC patients [13–16]. Considering the ubiquitous role of cytokines in human diseases [17], subsequent studies investigated whether SC levels would be different between OC patients and individuals with inflammatory or benign oral mucosa conditions [18–20]. These studies supported the association between SC and OC, corroborating with the hypothesis that SC are potential OC diagnosis biomarkers.

Despite of that, there is still no consensus on the discriminatory power for each SC, or whether these cytokines are able to differentiate OC from OPMD and other inflammatory oral diseases. Therefore, the objective of this systematic review and meta-analysis is to identify the SC with potential diagnostic power for OC detection and to verify whether the levels of these SC are consistent among different studies. The results presented here are of great relevance for guiding future technical and clinical endeavors seeking the development of early stage OC biomarkers based on SC, which is an essential step towards treatment improvement for OC patients.

## Methods

This review was performed following the Cochrane Handbook for Systematic Reviews of Interventions (Version 5.1.0) [21], also registered and published at the International prospective register of systematic reviews (PROSPERO - n° CRD42018111397). Data search, screening and extraction were executed by two of the authors (MMAC and CBZ).

Data search was performed using the electronic databases PubMed and Cochrane Library, considering manuscripts published between 1950 and 2019 and 1999 and 2019, respectively. The searching terms used were: “*cytokine”*, “*oral cancer”* and *“saliva”*, and also combined with “*interleukin” or “interferon”.* The searching period was from September 3rd, 2018 to January 31st, 2019.

The resulting manuscripts were initially screened by title and abstract, followed by a full-text analysis. Duplicates were checked manually and removed by two of the authors (MMAC and CBZ). Manuscripts were then selected based on the following inclusion criteria:

- Publication type: Peer-reviewed original articles, published in the English language;
- Study design: Case-control studies with human subjects
- Exposure of interest: Participants enrolled in the “case” group diagnosed with OC and “controls” corresponding to healthy subjects. For both groups, SC should have been measured in its protein form.
- Method of sampling: SC measured using enzyme-linked immunosorbent assay (ELISA).
- Research question: Studies comparing SC levels between OC patients, non-cancer control subjects and patients with OPMD or leukoplakia.

In case of discordance about the eligibility criteria, a third author (ARB) was consulted.

### Data extraction

A database was created to organize the information from the selected publications, including author details, year of publication, number of patients, and mean SC expression values with standard deviation or standard error of the mean. Two authors (MMAC and CBZ) analyzed the data and a third author (ARB) re-evaluated all the information.

The selected articles were further evaluated considering the following aspects: whether the period of sample collection was reported; if the study was prospective; if the techniques for saliva collection, storage, and analysis were standardized and properly reported.

### Data analysis

The data used for the meta-analysis were sample size, mean and standard deviation of the expression of SC in healthy controls, OC patients and patients with OPMD, including leukoplakia. Studies lacking any of these information were excluded. All data for the standard errors of the mean (SEM) were converted to standard deviations (SD) and all measurements were converted to picograms of protein per milliliter of saliva (pg/mL), and studies were excluded when this conversion was not possible. Measurements that were observed in single studies were also excluded from the meta-analysis because of the impossibility to estimate a combined effect. All these findings, however, were included in the qualitative analysis.

The random effects meta-analysis was conducted using the “meta” package in R. The standard mean difference (SMD) was estimated using the Hedges’ g, and the in-between study variance (τ^2^) was calculated using the DerSimonian-Laird estimator. All in-text data is represented as SMD followed by 95% confidence intervals (CI).

## Results

**Fig. 1:**
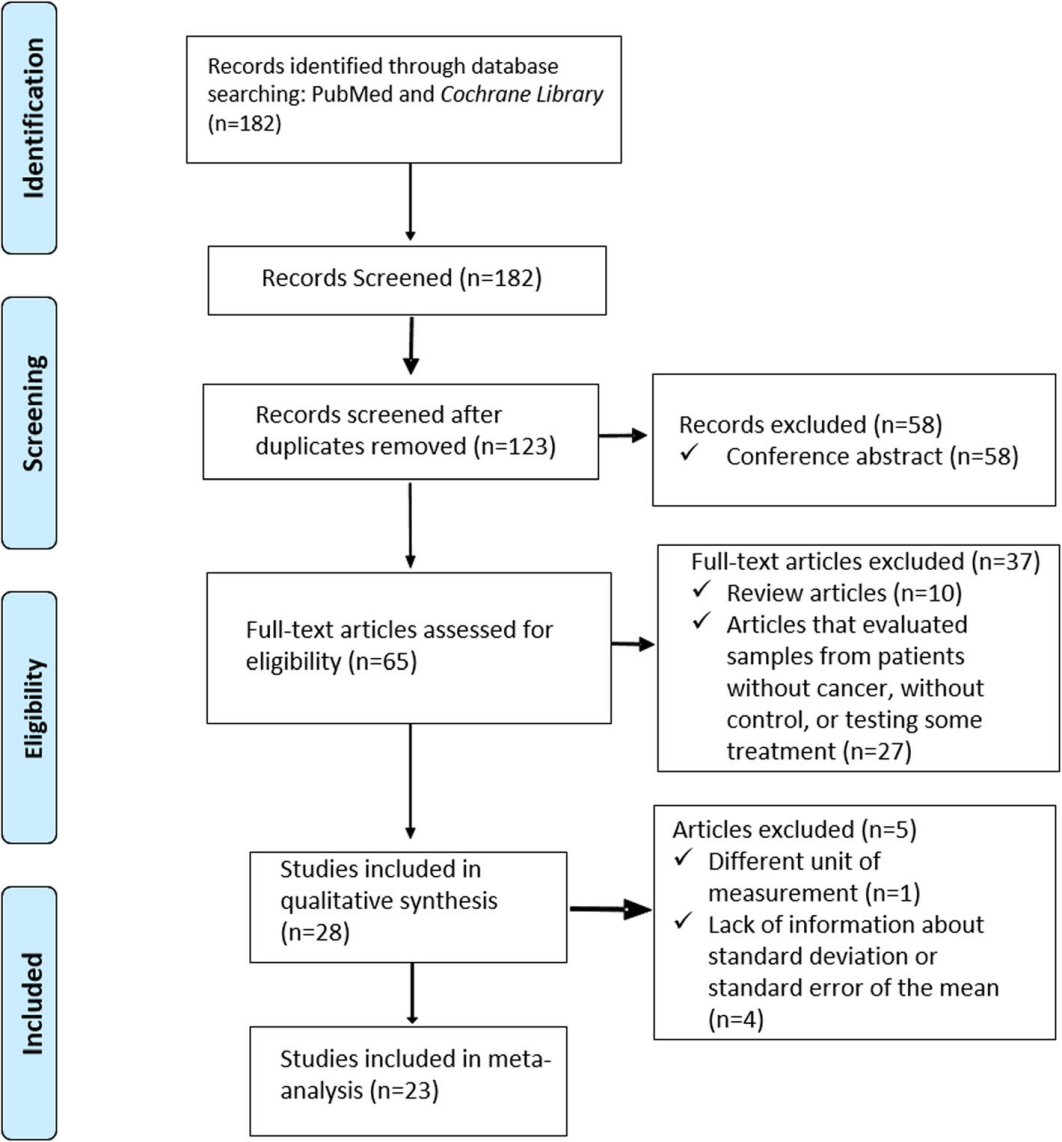
PRISMA flow diagram of study selection process (Moher, Liberati, Tetzlaff, Altman, & PRISMA Group, 2009)

The initial search in electronic databases PubMed (1950–2019) identified 182 studies. After screening, a total of 28 studies were included into our qualitative analysis (Fig. 1). Among the selected articles, Polz-Dacewicz et al. (2016) reported SC concentrations in nanograms of protein per milliliter of saliva (ng/mL), which were converted to picograms of protein per milliliter of saliva (pg/mL) aiming to standardize their findings with the other studies. Gonçalves et al. (2015) was not included in the meta-analysis because the measurements were reported in picograms of SC per milligram of total salivary protein (pg/mg). Four studies were excluded because of lacking information concerning SD or SEM.

**Fig. 2:**
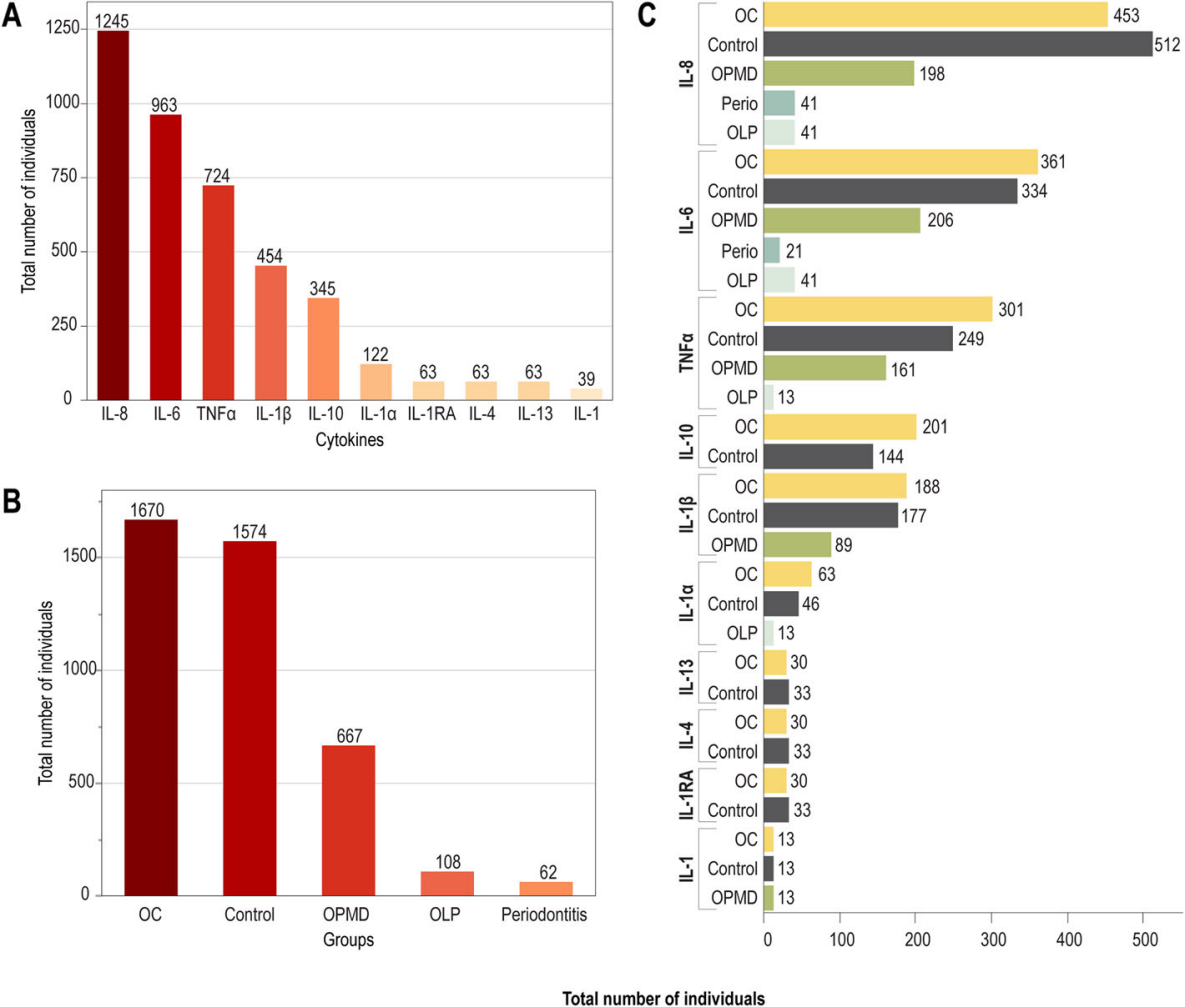
Sample size distribution among the investigated studies according to: A) Salivary cytokines assessed. IL-8, IL-6, TNFα and IL-1β were the cytokines investigated in the biggest number of patients; B) Patient clinical groups (OC = Oral Cancer; Control = healthy individuals; OPMD = Oral Potentially Malignant Disorders, OLP = Oral Lichen Planus). Healthy patients were the most numerous among control groups, followed by individuals with OPMD; C) Salivary cytokines assessed per each patient clinical group

The selected 28 studies were all published between 2004 and 2018, from 12 different countries and the sample size ranged from 18 to 300 patients. The control groups were composed by healthy individuals, patients with OPMD, oral lichen planus (OLP), or periodontitis (Tables 1, 2, 3, 4, 5 and 6). The total number of patients, combining the 28 studies, considering each SC evaluated and each study group are described in the Fig. 2. IL-8 was investigated in 1245 individuals, followed by IL-6 and Tumor Necrosis Factor α (TNFα), investigated in 963 and 724 individuals, respectively. In total, the SC identified in the frequency of appearance in these studies were IL-8 (50%), IL-6 (50%), TNF-α (28.6%), IL-1β (21.4%), IL-10 (17.9%), IL-1α (10.7%) and IL-1, IL-1RA, IL-4 and IL-13 (3.6% each) (Fig. 2a). Among the studied groups in the 28 selected studies, OC had the higher number of individuals included (1670), followed by 1574 healthy subjects, 667 patients with OPMD, 108 with OLP and 62 with periodontitis (Fig. 2b). The number of occurrences of each SC for each condition is summarized in Fig. 2c.

**Table 1:** Salivary interleukin 8 in oral cancer

Cytokine	Author	Year	Groups	n of cases	Mean ± SD (pg/ml)	Statistic test	***p*** Value	AUC
IL 8	SAHEBJAMEE M. et al.	2008	Oral Cancer	9	1093.7 ± 1089.0	Kolmogorov-Smirnov Test and Mann-Whitney U	< 0.05	–
			Control	9	700.7 ± 1031.5			
	ELASHOFF, D. et al.	2012	Oral Cancer - cohort 4	36	2563.0 ± 2179.0	Mann–Whitney U test and area under the curve (AUC)	< 0.05	0.680
			Control - cohort 4	54	808.0 ± 1132.0			
			Oral Cancer - cohort 5	31	2140.0 ± 2282.0		< 0.05	
			Control - cohort 5	70	739.0 ± 1002.0			
	ARELLANO-GARCIA, M.E. et al.	2008	Oral Cancer	40	3347.7 ± 2929.0	Student t-test, Pearson correlation coefficient; ROC curve and area under curve	With single-plex: 0.02 With multiplex: 0.04	0.820 (Sensitivity = 87.5% Specificity = 64.3%)
			Oral Cancer Control	42	759.4 ± 563.0			
			Periodontitis	10	818.8 ± 228.4			
			Periodontitis Control	10	589.2 ± 370.3			
	TANN, W. et al.	2007	Oral cancer	20	1252.0 ± 456.0	T-test and area under curve	< 0.00001	0.837
			Control	20	577.0 ± 355.0			
	KATAKURA A. et al.	2007	Oral Cancer	19	720.0	Two-tailed t-tests	< 0.05	–
			Control	20	250.0			
	RAJKUMAR, K. et al.	2014	Oral Cancer	100	1091.7 ± 167.1	Shapiro-Wilk’s test, Kruskal-Wallis analysis, Mann-Whitney U test, Spearman rank test, Receiver operator characteristic (ROC) curve and area under curve	< 0.05	PML x OSCC = 0.971 (95%CI = 0.953–0.990; *p* < 0.0001)
			Premalignant	100	650.4 ± 207.3			
			Control	100	349.6 ± 115.1			
	CHENG, L.Y.S. et al.	2014	Oral Cancer	5	1525.3 ± 1123.9	Kruskal-wallis test Mann-Whitney U test (post hoc)	< 0.001	–
			Chronic periodontitis	21	738.8 ± 394.0			
			Disease active – Oral lichen planus	15	1328.4 ± 731.8			
			Disease inactive – Oral lichen planus	13	1083.1 ± 646.2			
			Control	21	890.8 ± 563.2			
**Cytokine**	**Author**	**Year**	**Groups**	**Sample (n)**	**Mean ± SD (pg/ml)**	**Statistic test**	**P Value**	**AUC**
IL 8	PUNYANI, S.R.; SATHAWANE, R.S.	2013	Oral Cancer	25	1718.6 ± 668.3	Scheffe’s analy- sis and two-tailed independent samples t test.	p < 0.0001	–
			Oral Precancer and Oral leukoplakia	25	299.5 ± 158.2			
			Control	25	210.1 ± 142.3			
	LEE, L.T. et al.	2018	Oral Cancer	41	2060.3 ± 1796.5	Kolmogorov-Smirnov and Mann-Whitney	< 0.001	0.783 (Sensitivity: 65.85% Specificity: 79.17% *p* = 0.0002)
			Control	24	907.0 ± 833.4			
	RHODUS, N.L. et al.	2005b	Oral lichen planus	13	2492.0 ± 664.7	One-way ANOVA, Student-Newman- Keuls q-test; t-test.	< 0.001	–
			Oral Cancer	13	4082.8 ± 752.3			
			Control	13	1507.2 ± 398.5			
	MAIE, A. R. et al.	2004	Oral Cancer	32	720.0	t-test, receiver operating characteristic (ROC) analyses	< 0.001	0.978 (Sensitivity: 86% Specificity: 97%)
			Control	32	250.0			
	KHYANI, I.A.M. et al.	2017	Oral Cancer	35	873.6	Pearson Chi-Square test, one-way ANOVA test, Post Hoc. Dunnet t-test	< 0.001	–
			Control	35	52.1			
			Premalignant	35	305.0			
	RHODUS, N.L. et al.	2005 a	Oral Cancer	13	3154.1 ± 1023.2	Not described	< 0.001	–
			Control	13	1580.7 ± 789.0			
			Premalignant	13	1918.2 ± 899.1			
	GLEBER-NETTO, FO. et al.	2016	Oral Cancer	60	283.7 ± 262.3	ANOVA (Kruskal - Wallis Test); Wilcoxon Two - Sample test	< 0.0001	OPMD vs. Controls = 0.467 OSCC vs. Controls = 0.449 OSCC vs. OPMD = 0.518
			Malignant pontentialy injuries	60	140.3 ± 155.1			
			Control	60	127.8 ± 110.8

**Table 2:** Salivary interleukin 6 in oral cancer

Cytokine	Author	Year	Groups	Sample (n)	Mean ± SD (pg/ml)	Statistic test	P Value	AUC
IL-6	PANNEER SELVAM N. and SADAKSHARAM J.	2015	Oral Cancer	25	132.9 ± 59.1	ANOVA and Kruskal-Wallis	< 0.001	–
			Control	25	9.7 ± 12.8			
			Leucoplakia	25	43.0 ± 52.1			
	JURETIĆ M. et al.	2013	Oral Cancer	19	0.707 ± 0.234	Kruskal-Wallis and Mann-Whitney	< 0.001	–
			Control	19	0.002 ± 0.002			
			Pre-malignant	19	0.431 ± 0.217			
	SAHEBJAMEE M. et al.	2008	Oral Cancer	9	40.9 ± 79.5	Kolmogorov-Smirnov Test and Mann-Whitney U	< 0.05	–
			Control	9	2.5 ± 1.3			
	ZHANG S. et al.	2017	Oral Cancer	40	4.8 ± 1.0	Unpaired t-test	< 0.01	–
			Control	20	1.3 ± 0.050			
	DINESHKUMAR T. et al.	2016	Oral Cancer	100	178.0 ± 28.3	Nonparametric Mann–Whitney U tests and ROC curve analysis	< 0.05	0.900 (Sensitivity = 99.0% Specificity = 96.0%)
			Control	100	10.3 ± 6.7			
			Pre-malignant	50	35.3 ± 14.3			
			Condition Premalignant	50	38.3 ± 12.3			
	KATAKURA A. et al.	2007	Oral Cancer	19	86.5	Two-tailed t-tests	< 0.05	–
			Control	20	–			
	BAGAN, L. et al.	2015	Oral Cancer	20	435.0 ± 142.1	Kruskal–Wallistest, Mann–Whitney U test, Spearman’s rank correlation coefficient	< 0.01	–
			Control	20	33.4 ± 38.9			
			Proliferative verrucous leukoplakia	20	151.6 ± 129.3			
	SATO, J. et al.	2010	Oral Cancer	29	20.1 ± 36.3	Mann-Whitney U test, Wilcoxon rank sum test, Spearman correlation coeficient	< 0.003	–
			Control	19	0.6 ± 0.8			
	KHYANI, I.A.M. et al.	2017	Oral Cancer	35	61.2	Pearson Chi-Square test, one-way ANOVA test, Post Hoc. Dunnet t tes	< 0.001	–
			Control	35	–			
			Pre-malignant	35	217.8			
IL-6	RHODUS, N.L. et al.	2005 a	Oral Cancer	13	88.2 ± 43.2	Not described	< 0.001	–
			Control	13	1.4 ± 1.0			
			Pre-malignat	13	70.8 ± 24.3			
	CHENG, L.Y.S. et al.	2014	Oral Cancer	5	178.4 ± 172.3	Kruskal-wallis test Mann-Whitney U test (post hoc)	< 0.001	–
			Chronic periodontitis	21	5.8 ± 4.0			
			Disease active – Oral lichen planus	15	20.7 ± 22.3			
			Disease inactive – Oral lichen planus	13	8.1 ± 8.0			
			Control	21	4.9 ± 8.8			
	LEE, L.T. et al.	2018	Oral Cancer	41	198.3 ± 303.8	Kolmogorov-Smirnov and Mann-Whitney	< 0.001	0.823 (Sensitivity: 82.9% Specificity: 70.3% p < 0.0001)
			Control	24	69.2 ± 272.0			
	BRAILO, V. et al.	2012	Oral Cancer	28	129.0 ± 66.3	Smirnoff Kolmogorof and Kruskal Wallis	< 0.012	–
			Leukoplakia	29	18.0 ± 5.2			
			Control	31	16.0 ± 3.9			
	RHODUS, N.L. et al.	2005 b	Oral lichen planus	13	148.1 ± 21.3	One-way ANOVA, Student-Newman- Keuls q-test and teste-t	< 0.0001	–
			Oral Cancer	13	198.2 ± 47.7			
			Control	13	2.3 ± 0.7

**Table 3:** Salivary TNFα in oral cancer

Cytokine	Author	Year	Groups	Sample (n)	Mean ± SD (pg/ml)	Statistic test	P Value	AUC
TNF – α	JURETIĆ M. et al.	2013	Oral Cancer	19	0.739 ± 0.176	Kruskal-Wallis and Mann-Whitney	< 0.001	–
			Control	19	0.013 ± 0.033			
			Premalignant	19	0.601 ± 0.178			
	RHODUS, N.L. et al.	2005 a	Oral Cancer	13	28.9 ± 14.6	Not described	< 0.01	–
			Premalignant	13	10.5 ± 7.4			
			Control	13	3.0 ± 1.0			
	SAHEBJAMEE, M. et al.	2008	Oral Cancer	9	35.2 ± 51.8	One Sample Kolmogorov-Smirnov test and Mann-Whitney U	< 0.05	–
			Control	9	4.1 ± 2.1			
	LEE, L.T. et al.	2018	Oral Cancer	41	27.7 ± 30.9	Kolmogorov-Smirnov and Mann-Whitney	< 0.001	0.749 (Sensitivity: 39.02% Specificity: 100% *p* = 0.0001)
			Control	24	8.6 ± 7.3			
	POLZ-DACEWICZ, M. et al.	2016	Oral Cancer	78	23,100.0	Pearson Chi-Square tests. Mann-Whitney and Kruksal-Wallis	0.00002	–
			Control	40	11,300.0			
	BRAILO, V. et al.	2012	Oral Cancer	28	34.0 ± 21.6	Smirnoff Kolmogorof and Kruskal Wallis	0.126	–
			Leukoplakia	29	30.1 ± 3.01			
			Control	31	38.0 ± 3.23			
	KRISHNAN, R. et al.	2014	Cancer	100	311.9 ± 95.3	Shapiro Wilk. Kruskal-Wallis e Mann-Whitney U	Cancer x Premalignant = *p* < 0.05 Cancer x Control = *p* < 0.001	0.981 (95%CI: 0.968–0.995)
			Leukoplakia	50	180.1 ± 52.4			
			Premalignant	50	166.5 ± 49.4			
			Control	100	4.5 ± 2.5			
	RHODUS, N.L. et al.	2005b	Oral lichen planus	13	74.2 ± 38.3	One-way ANOVA, Student-Newman- Keuls q-test; teste-t.	< 0.0001	–
			Oral Cancer	13	103.6 ± 61.7			
			Control	13	3.4 ± 2.1

**Table 4:** Salivary Interleukin 1β in oral cancer

Cytokine	Author	Year	Groups	Sample (n)	Mean ± SD (pg/ml)	Statistic test	P Value	AUC
IL-1β	ELASHOFF, D. et al.	2012	Oral Cancer – cohort 5	31	293 ± 396	Mann–Whitney U test and area under the curve (AUC)	< 0.05	0.570
			Control - cohort 5	70	169 ± 202			
	ARELLANO-GARCIA, M.E. et al.	2008	Oral Cancer	40	591.5 ± 618.7	Student t-test, Pearson correlation coefficient; ROC curve and area under curv	With single-plex: 0.03 With multiplex: 0.04	0.840 (sensitivity = 63.9%; specificity = 100%)
			Control Oral Cancer	42	79.6 ± 578			
	KATAKURA A. et al.	2007	Oral Cancer	19	158.9	Two-tailed t-tests	< 0.05	–
			Control	20	14.1			
	GLEBER-NETTO, FO. et al.	2016	Oral Cancer	60	101.0 ± 113.0	ANOVA (Kruskal - Wallis Test); Wilcoxon Two - Sample test	Oral Cancer x Control = p < 0.01; Oral Cancer x Maligmant Potencial = *p* < 0.004	OPMD vs. Controls = 0.542 OSCC vs. Controls = 0.721 OSCC vs. OPMD = 0.569
			Malignant potentialy injuries	60	39.7 ± 28.0			
			Control	60	48.1 ± 42.0			
	LEE, L.T. et al.	2018	Oral Cancer	41	391.4 ± 540.4	Kolmogorov-Smirnov and Mann-Whitney	0.002	AUC: 0.729 (Sensitivity = 60.98%; Specificity = 79.17%; *p* = 0.0004)
			Control	24	132.5 ± 175.8			
	BRAILO, V. et al.	2012	Oral Cancer	28	906.0 ± 62.2	Smirnoff Kolmogorof and Kruskal Wallis	0.000	–
			Leukoplakia	29	143.0 ± 54.7			
			Control	31	354.0 ± 61.4

**Table 5:** Salivary interleukin 10 in oral cancer

Cytokine	Author	Year	Groups	Sample (n)	Mean ± SD (pg/ml)	Statistic test	P Value	AUC
IL-10	HAMZAVI, M. et al.	2014	Oral Cancer	30	11.8 ± 10.7	Kolmogorov-Smirnov test. Mann-Witney, Kruskal-Wallis and Chi-Square tests	0.619	–
			Control	24	10.0 ± 6.0			
	AZIZ, S. et al.	2015	Oral Cancer	30	4.4 ± 4.3	Teste - t. One way ANOVA e LSD Post hoc	0.004	–
			Control	33	1.7 ± 1.3			
	LEE, L.T. et al.	2018	Oral Cancer	41	14.9 ± 20.2	Kolmogorov-Smirnov and Mann-Whitney	0.355	–
			Control	24	9.9 ± 8.5			
	POLZ-DACEWICZ, M. et al.	2016	Oral Cancer	78	5.9	Pearson Chi-Square tests. Mann-Whitney and Kruksal-Wallis	0.00002	–
			Control	40	2.5			
	GONÇALVES, A.S. et al.	2015	Oral Cancer	22	0.037*	Shapiro-Wilk Mann- Whitney Fisher and Pearson Chi-Square tests.	0.038	–
			Control	23	0.027*

**Table 6:** Salivary interleukin 1α in oral cancer

Cytokine	Author	Year	Groups	Sample (n)	Mean ± SD (pg/ml)	Statistic test	P Value	AUC
IL-1α	SAHEBJAMEE M. et al.	2008	Oral Cancer	9	201.7 ± 178.8	One Sample Kolmogorov-Smirnov test and Mann-Whitney U	< 0.05	–
			Control	9	178.2 ± 170.7			
	LEE, L.T. et al.	2018	Oral Cancer	41	995.7 ± 932.6	Kolmogorov-Smirnov and Mann-Whitney	0.625	–
			Control	24	1054.6 ± 1584.9			
	RHODUS, N.L. et al.	2005b	Oral lichen planus	13	293.6 ± 86.8	One-way ANOVA, Student-Newman- Keuls q-test; teste-t.	< 0.001	–
			Oral Cancer	13	370.5 ± 29.7			
			Control	13	135.9 ± 28.4

**Fig. 3:**
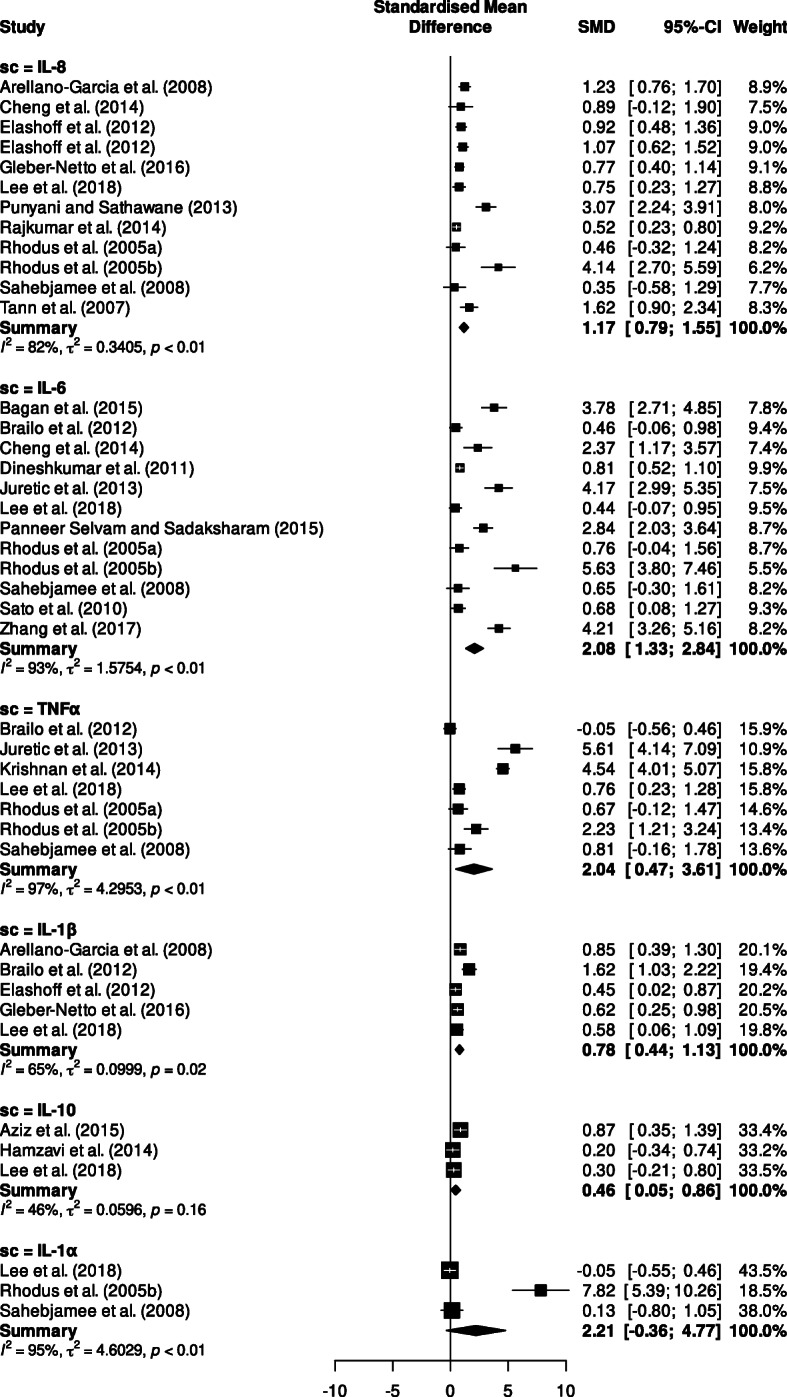
Random-effects meta-analysis of the salivary cytokine levels in oral cancer patients in comparison to healthy controls (SMD = 0)

The meta-analysis comparing the concentration of SC in OC patients versus healthy controls showed a significant increase in the level of IL-8 (standardized mean difference (SMD) = 1.77; 95% CI 0.79 to 1.55), IL-6 (SMD = 2.08; 95% CI 1.33 to 2.84), TNF-α (SMD = 2.04; 95% CI 0.47 to 3.61), IL-1β (SMD = 0.78; 95% CI 0.44 to 1.13), and IL-10 (SMD = 0.46; 95% CI 0.05 to 0.86) in the cancer group. IL-1α was the only SC that did not present a significant difference (SMD = 2.21; 95% CI − 0.36 to 4.77) (Fig. 3). IL-1, IL-1RA, IL-4 and IL-13 were excluded from the analysis because there was just a single observation for each.

Most likely due to variations in experimental procedures for saliva collection and SC quantification, in-between studies heterogeneity was high in most cases. When comparing OC vs. controls, heterogeneity was non-significant only for IL-10 (I^2^ 46%, *p* = 0.16).

### Interleukin-8

**Fig. 4:**
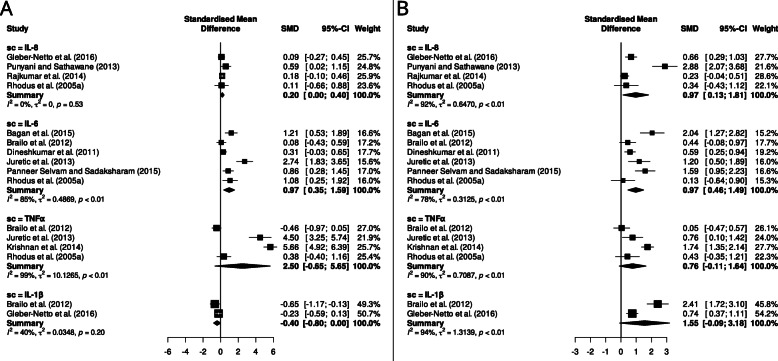
Random-effects meta-analysis of salivary cytokine levels in patients with oral potentially malignant disorders (OPMD) in comparison to healthy controls (**a**) and in patients with oral cancer in comparison to individuals with OPMD (**b**). In both cases, SMD = 0 for the second group referred

IL-8 levels in OC patients were compared to controls in 14 articles. The study with the smaller population of OC included five patients (Cheng, S. et al. 2014), and the largest one evaluated SC concentration in 100 OC patients (Rajkumar, K. et al. 2014). Among these 14 articles, 14 groups of OC, 5 groups of OPMD patients, 2 groups of OLP and 2 groups of periodontitis patients were evaluated. In all these studies, IL-8 levels were reported to be significantly different between OC patients and controls. The values of IL-8 in control group varied from 52.1 to 1580.7 pg/mL; for the premalignant group, values between 140.3 and 1918.2 pg/mL were found; and in the OC group, the values were between 283.7 and 4082.8 pg/mL [14, 22–34]. Out of these 14 studies, 11 were eligible for meta-analysis, and the combined effect was significant, indicating an increase in the concentration of this SC in OC patients comparison to controls (SMD = 1.77; 95% CI 0.79 to 1.55) (Fig. 3). However, when comparing IL-8 salivary concentration in OPMD patients against healthy controls, it was not significant with a bordering value (SMD = 0.20; 95% CI 0.00 to 0.40), and heterogeneity was very low (I^2^ = 0%, *p* = 0.53) (Fig. 4a). IL-8 concentration in OC patients was significantly higher than what was observed in OPMD patients (SMD = 0.97; 95% CI 1.81 to 0.13), but heterogeneity was very high (I^2^ = 92%, *p* <  0.01) (Fig. 4b).

### Interleukin-6

Interleukin-6 (IL-6) was evaluated in 14 articles, and in all of them the amount of IL-6 was reported to be statistically higher in OC patients compared to the control group. The population of the studies varied from 9 subjects in the smallest study, to 100 subjects in the largest one. Five articles only compared OC patients with a control group of non-cancer people, while 07 articles also compared the OC with OPMD, and one article compared OC patients with a non-cancer group with periodontitis. The IL-6 values for healthy subjects varied from 0 to 16.0 pg/mL in ten of the articles [18, 20, 22, 26, 31, 34–38], while in two studies it was found in higher values (33.4 and 69.2 pg/mL) [19, 39]. The difference in IL-6 salivary concentration between OC patients and healthy individual was also observed in the meta-analysis, that included 12 studies (SMD = 2.08; 95% CI 1.33 to 2.84). However, heterogeneity was significant (I^2^ = 93%, *p* <  0.01) (Fig. 3).

For the OPMD group, values between 0.431 and 35.3 pg/mL were reported in four articles [20, 31, 35, 37] and higher values, between 43.0 and 217.8 pg/mL were found in another five studies [18, 19, 25, 26, 34]. In the OC groups, the values were from 0.707 to 435.0 pg/mL [14, 18–20, 22, 25, 26, 31, 34–39], and the values were higher than 80.0 pg/mL in nine studies [14, 18–20, 26, 31, 34, 37, 39]. Meta-analysis showed that IL-6 concentration was significantly higher in OPMD patients in comparison to healthy controls (SMD = 0.97; 95% CI 0.35 to 1.59) (Fig. 4a), while OC patients showed significantly higher IL-6 concentrations in comparison to the OPMD group (SMD = 0.97; 95% CI 1.49 to 0.46) (Fig. 4b). In both cases, heterogeneity was high (I^2^ = 85 and 78%, respectively and *p* < 0.01 in both comparisons).

### Tumor necrosis factor-α (TNF-α)

TNF-α was analyzed in 8 articles and it was found in a significantly higher amount in OC patients compared to controls in 7 of these studies. The study population varied from 9 to 100 subjects. Three studies only compared OC patients with a healthy control group [22, 39, 40], while 5 articles compared the OC patients with subjects with OPMD [20, 26, 34, 35, 41]. The TNF-α values for the control groups were from 0.013 to 11,300.0 pg/mL. For OC patients, the TNF-α values varied from 0.739 to 23,100.0 pg/mL [13, 26, 34, 35, 39–42]. Meta-analysis comparing salivary TNF-α concentration in OC patients against control subjects included seven studies and indicated a significant increase (SMD = 2.04; IC 95% 0.47 to 3.61) (Fig. 3). However, heterogeneity, as measured by Higgins’ I^2^, reached 97% (*p* < 0.01). Salivary TNF-α concentrations were not significantly different in OPMD patients in comparison to controls nor in OC patients in comparison to OPMD (SMD = 2.50; 95% CI − 0.65 to 5.65 and SMD = 0.76; 95% CI − 0.11 to 1.64, respectively). For both summaries, heterogeneity was high (I^2^ = 99 and 90%, respectively, and p < 0.01 in both cases) (Fig. 4a-b).

### Interleukin-1β

IL-1β was analyzed in 6 articles and a significant difference in salivary concentration was found between OC patients and control subjects in all studies. The number of subjects included in the studies varied from 28 to 60. This difference was also observed in the meta-analysis, that included five studies (SMD = 0.78; 95% CI 0.44 to 1.13). Heterogeneity between studies was low (I^2^ 65%, *p* = 0.02) (Fig. 3). Two studies compared OC patients with both OPMD patients and healthy controls [20, 43], and 4 studies compared OC patients with only healthy subjects [23, 39]. The IL-1β values in the control group varied from 14.1 to 354.0 pg/mL. In the OPMD group the variation was between 39.6 to 143.0 pg/mL, and in the OC patients it varied from 101.0 to 906.0 pg/mL [14, 23, 28, 39, 42, 43]. Meta-analysis showed that IL-1β concentration in OPMD patients was not significantly different from controls with a bordering value, in fact being the only SC showing a tendency of reduction in this condition (SMD = − 0.40; 95% CI − 0.80 to 0.00). Heterogeneity was once again low (I^2^ = 40%, p = 0.02) (Fig. 4a). IL-1β concentration in OC patients in comparison to the OPMD group was not significantly different (SMD = 1.55; IC 95% -0.09 to 3.18) and with high heterogeneity (I^2^ = 94%, *p* < 0.01) (Fig. 4b).

### Interleukin-10

IL-10 was evaluated in 5 articles and in three of them was observed statistical difference in OC patients compared do control subjects. The studies evaluated from 22 to 78 subjects per group. All articles compared OC patients to control subjects, not including OPMD patients. The salivary IL-10 values were between 0.027 and 10.0 pg/mL in control groups, and between 0.037 and 14.9 pg/mL in OC patients [39, 40, 44–46]. Meta-analysis included three studies, and the combined effect indicates an increase in the salivary concentration of IL-10 in OC patients in comparison to healthy subjects (SMD = 0.46; 95% CI 0.05 to 0.86). Heterogeneity of this analysis was low (I^2^ = 46%, *p* = 0.16) (Fig. 3).

### Interleukin-1α

IL-1α was evaluated in 3 articles, two compared OC patients with healthy controls [22, 39] and one study also compared with OLP [34] (Fig. 3). The studies included from 9 to 41 subjects per group. Two of these articles presented results with significant difference between OC and controls. The IL-1α quantification varied from 135.9 to 1054.6 pg/mL in the control groups, was of 293.6 pg/mL in the OLP group, and between 201.7 and 995.7 pg/mL in OC patients [22, 34, 39]. Results from meta-analysis showed a non-significant difference between salivary levels of IL-1α of OC patients in comparison to controls (SMD = 2.21; 95% CI − 0.36 to 4.77), even though the analysis had a high heterogeneity (I^2^ = 95%, *p* < 0.01) (Fig. 3).

### Other cytokines

Due to the lack of studies to draw comparisons, the remaining cytokines were included only in a qualitative analysis.

IL-1RA was found in one study that compared OC patients (*n* = 30) with healthy controls (*n* = 33) (Fig. 3). The salivary concentration of IL-1RA in the OC group was of 2831.6 pg/mL, and 1949.2 pg/mL in control group, not showing significant difference between them [46].

IL-1 (without specification of the subunit) was studied in one article, comparing OC patients with patients with OPMD and a group of healthy controls (Fig. 3), each group was composed of 13 individuals. Differences were found in the concentration of salivary IL-1 between groups. The IL-1 value found in OC patients was of 454.4 pg/mL, in premalignant lesions was of 255.1 pg/mL, and, in healthy controls, of 173.2 pg/mL [26].

IL-4 was studied in one article comparing OC patients (n = 30) with healthy subjects (n = 33) (Fig. 3). OC patients present 1.2 pg/mL of IL-4 in saliva, while healthy subjects 1.0 pg/mL, without statistical difference between groups [46].

IL-13 also was analyzed in one study, comparing the same groups as the previous one (Fig. 3). In OC patients the IL-13 level was 0.760 pg/mL, and in the control groups 0.230 pg/mL, showing a difference statistically significant [46].

## Discussion

Pro-inflammatory and anti-inflammatory cytokines are produced and released in the tumor microenvironment by tumor and immune cells [47]. Studies with saliva have shown increased cytokine levels in cancer patients, irrespective of pro-inflammatory or anti-inflammatory cytokine activity [33, 48].

In the studies analyzed in this review different pro-inflammatory and anti-inflammatory cytokines were evaluated (pro-inflammatory: IL-1, IL-1α, IL-1β, IL-RA, IL-6, IL-8 and TNFα; anti-inflammatory: IL-4, IL-10 e IL-13). The most studied cytokines were IL-8 and IL-6. The salivary levels of IL-8, IL-6, TNF-α, IL-1β and IL-1α were significantly higher in OC patients compared to healthy controls. Only one anti-inflammatory SC (IL-10) was evaluated by meta-analysis, and did not presented significant difference between OC and NCC. Furthermore, the levels of salivary IL-8, IL-6, TNF-α and IL-1β were higher in OC patients compared to PMOL, and comparing the NCC vs PMOL patients, was observed a significant increasing in salivary levels of IL-6 and TNF-α in PMOL group.

Our study showed that some SC show great potential as diagnostic biomarkers for OC, serving as a non-invasive alternative for early diagnosis. The increased concentration of certain SC, most notably IL-8, IL-6 and TNF-α, could be used as biomarkers for OC, as their concentration was significantly higher in patients with the disease in comparison to healthy subjects. However, the levels of these SC are also increased due to other conditions, such as OPMD. Thus, it is important to determine how different is this increase when comparing the two clinical conditions. It was observed that the concentration of IL-8 and IL-6 in OC patients was significantly higher in comparison to OPMD patients, even with a significant increase in comparison to healthy subjects, making these two of the most promising SC as reliable diagnostic tools. None of the other SC included in the meta-analysis showed a clear difference in concentration between OC and OPMD patients, even though differences were observed when comparing patients with OC patients and healthy subjects, (namely, TNF-α, IL-1β and IL-10). This suggests good potential to determine OC, even though evidence for these SC is more limited.

There are still technical and biological issues that must be addressed for a final and definitive evaluation of the clinical value of these biomarkers. Even though all studies analyzed in this review used the ELISA, considered as the most accurate technique to measure proteins in biofluids [16], one of the main limitation of the results is the high heterogeneity of the results collected, even in the control groups. Even though this variability limits the interpretation of the meta-analysis, it favors the discussion about the development and implementation of standard operating procedures (SOP) in the field of salivary biomarkers, leading to its technical development and facilitating its translational path. With more standardized methods of saliva collection and storage and SC quantification, thresholds could be identified to distinguish between different oral conditions.

Even though the studies compared patients with OC with different control groups, from healthy subjects to OPMD and inflammatory conditions, the selection of non-cancer individuals was heterogeneous between the studies, making difficult to carry out direct comparisons between them. This is another point to be observed and standardized in future studies, since the demographic and clinical characteristics of the controls and disease-inflicted subjects must show correspondence.

Although the relative expression of SC demonstrated a good potential to distinguish OC patients from non-cancer subjects, and the increased levels of these cytokines in OC patients are consistent, the salivary biomarkers should be tested in combination with clinical examination [49]. The use of these biomarkers must be improved to be used specifically in at-risk populations as an auxiliary method for screening and early diagnosis.

An important limitation of the studies we evaluated is the absence of a group of early stage OC patients. Most studies did not separate patients according to disease stage, which limits the extrapolation of these findings to early cancer diagnosis [50]. Some studies showed a positive correlation between cytokine levels disease stage [33]. In this way, it is important that the diagnostic performance of SC is evaluated in the group with early stage disease.

Another limitation is the ELISA kits used to measure the cytokines in these studies, which are reagents label as “Research Use Only”, indicating that there is no strict regulation on the technical characteristics of the tests. Thus, the comparison of results between different studies is limited, since the variation between brands or even lots can lead to variations in results. For the continuity of the validation process of these SC as biomarkers for OC it is necessary the development of studies that consider the variability of the disease presentation, the measurement of the cytokines in a controlled environment, and using reagents developed to clinical use. Moreover, so that possible biases are excluded, multicenter studies must be performed, using a larger samplez than the previous studies [15, 51]. And for the results to be reliable, it is of fundamental importance that an international standardization be validated for both saliva collection and SC measurements.

## Conclusions

In this review, we found 28 articles that evaluated the concentration of 10 different pro and anti-inflammatory SC in OC patients. IL-8 and IL-6 were the most studied ones, and in all articles these salivary cytokines were found at higher levels in OC patients compared to healthy controls and, in most cases, OPMD patients.

A meta-analysis with twenty-three studies showed that salivary levels of IL-8, IL-6, TNF-α, IL-1β and IL-1α are significantly higher in OC patients compared to healthy controls; the levels of salivary IL-8 and IL-6 are higher in OC patients compared to OPMD, and comparing the controls vs OPMD patients, it was observed a significant increase in salivary levels of IL-6 in OPMD group. These results suggest that, mainly the pro-inflammatory cytokines IL-8 and IL-6 can be explored in the future to determine the real potential as a biomarker for OC. Furthermore, it was found a big variability in the SC concentrations in the different studies, even when reporting the same quantification methodology.

In order to translate these biomarkers into the clinical practice, standardization of saliva collection and cytokines measurement process is required, as well as larger and multicentric studies.

## Data Availability

All data generated or analyzed during this study are available for consultation and can be requested from authors.

## Abbreviations

SC: Salivary cytokines
OC: Oral squamous cell carcinoma
OPMD: oral potentially malignant disorders
ELISA: enzyme-linked immunosorbent assay
OLP: oral lichen planus
IL-8: interleukin-8
IL-6: Interleukin-6
TNF-α: Tumor necrosis factorα
IL-1β: Interleukin-1β
IL-10: Interleukin-10
IL-1α: Interleukin-1α
IL-1RA: Interleukin-1RA
IL-4: Interleukin-4
IL-13: Interleukin-13
SMD: standardized mean difference
SOP: standard operating procedures
